# Supplementary material for: Maternal Fiber Intake and Perinatal Depression and Anxiety
Source: Nutrients. 2024 Jul 31;16(15):2484. doi: 10.3390/nu16152484 (PMC11313952; doi:10.3390/nu16152484)
Supplement: Supplementary file 1 [file nutrients-16-02484-s001.zip › nutrients-3056361-supplementary.pdf]

Table S1: characteristics of the studies included

Study Details			Mental Health Assessment Details						Dietary Assessment Details				Mental Health Outcomes
			Depression		Anxiety		Tool	Timing	FFQ	Timing	Recall period	Dietary Pattern	Key findings
Author & Year	Country	# Subjects Analyzed	Prenatal	Postpartum	Prenatal	Postpartum	Name of Assessment Tool	Gestational Weeks= W Postpartum Months=M	Name of FFQ (# of Food Groups)	Gestational Week (GW) Or Postpartum (M)	24 hours; Weeks, Months	DP categories or Intake of Food Groups	(p-value <0.05)
Vilela, 2015	**Brazil**	196			**x**	**x**	STAI-State	3 times:<br>20-26 W<br>30-36 W<br>4-6 M	Brazilian FFQ (19)	Once in 1st trimester	6M pre-conception	1) Common Brazilian<br>2) Healthy<br>3) Processed	Sig. Inverse association between Healthy DP and Common Brazilian DP and anxiety symptoms throughout.<br><br>No. Sig. association between processed pattern and anxiety symptoms
Vilela, 2014	**Brazil**	248	**x**				EPDS	3 times:<br>Once per trimester	Brazilian FFQ (18)	Once in 1st trimester	6M pre-conception	1) Common Brazilian<br>2) Healthy<br>3) Processed	Sig. inverse association between Healthy DP and depressive symptoms at all three points during pregnancy.<br><br>No Sig. association between Common Brazilian, Processed and symptoms of depression
Paskulin, 2017	**Brazil**	712		**x**		**x**	PRIME-MD	Two times<br>1M; 5M	Brazilian FFQ (19)	Once at<br>16-36 GW	Last 12M	1) Varied<br>2) Restricted<br>3) Common Brazilian	**Compared to the Varied DP**<br><br>↑ Brazilian DP = 43%↑ MDD PR<br>↓ fruit intake = 43% ↑ MDD PR<br>↑ sweets/sugar = 91% ↑ MDD PR<br><br>↓ beans = 40%↑ anxiety PR
Baskin, 2017	**Australia**	167	**x**	**x**			PDS	3 times:<br>1st trimester<br>2nd trimester<br>3rd trimester	Cancer Council of Victoria FFQ (34)	2nd & 3rd Trimester	Last 3M	1) Healthy<br>2) Unhealthy	Unhealthy Pattern @ 2nd Trimester, Predicted depression @ 2nd trimester ß: [+0.16 (0.02-0.30)] p-value <0.05
Galbally, 2021	**Australia**	442	**x**	**x**			**EPDS**	3 times:<br>3rd trimester.<br>6M; 12M	FFQ (8)	3times: 3rd trimester; 6M; 12M	Last month	Intake of Different FG in depressed & Untreated vs. On Antidepressant vs. Healthy	↑ EPDS Scores = ↓ intake of fruits & vegetables in the 3rd trimester<br><br>↑ EPDS = ↑ monthly takeaway meals<br><br>↑ takeaway meals in women with untreated depression compared to both antidepressant treated and healthy groups.
Shi, 2020	**China**	565		**x**			**EPDS**	?	?	1 time: early postpartum	1 Month Prior to Delivery	Intake of Different Food Groups in depressed vs. non depressed	↑ meat intake, ↓ vegetables, ↓ fruit, ↓ fish, ↓ poultry in women with postpartum depression

Cao, 2020	**China**	1659		**x**			**SDS**	One time: 6-12M	Semi Quantitative FFQ (13)	One time: 6-12M	3rd Trimester intake (3-Months)	Beverage DP<br>Vegetable DP<br>Cereal/Meat DP<br>Nut-Fruit DP<br>Egg DP<br>Seafood DP	↓ depression with higher adherence to Nut-Fruit DP [OR=0.74(0.57-0.95;p-value=0.016)] & Seafood DP [OR=0.75(0.58-0.98; p-value=0.033)].
Huang, 2021	**China**	17430	**x**				**SDS**	Two times: Early (<20W) & Late (35-38W)	FFQ (30)	One time: 24-28Wk	1 week	Varied DP (Reference)<br>Vegetable DP<br>Meat DP<br>Cereal DP<br>Milk DP<br>Fruits DP	Compared to Varied DP:<br>21%↓ in OR of depression in Vegetable DP<br>25%↓ in Milk DP<br>23%↓ in Fruits DP<br>Sig. Inverse association between Vegetable, Milk and Fruit DPs and SDS Scores
Okubo, 2012	**Japan**	865		**x**			**EPDS**	2 to 9 M postpartum	DHA (33)	2x. first @ 20Wks GA & 2-9M PP)	1 Month	Healthy<br>Western<br>Japanese	Only the 2nd quartile of Western DP associated with a sig. reduction in postpartum depression OR: 0.55(95%CI: 0.30-0.98) (adjusted model)<br><br>2nd quartile of Japanese DP associated with reduction in OR of PPD: OR: 0.52(95%CI:0.30-0.93) (Crude model)
Miyake, 2018	**Japan**	1744	**x**				**CES-D**	1 time: 5-39 W	DHQ (33)	1 time: 5-39W	1 Month	Healthy DP<br>Western DP<br>Japanese DP	Greater adherence to Healthy and Japanese DP associated with sig. 24%-52% ↓ in depression
Miura, 2020	**Japan**	92448	**x**		**x**		**SF8-HRQOL**	1 time: 1st Trimester	JECS (23)	1 time: 1st Trimester	Last 12 months	Western DP<br>Japanese DP<br>Unbalanced DP	Higher adherence to Japanese and unbalanced DPs associated with 20% ↑& 29%↑ OR of poor mental health
Teo, 2018	**Singapore**	490	**x**	**x**	**x**	**x**	**EPDS & STAI**	2 times: 26-28W & 3M-PP	3-day diary (84)	1 time @ 1M PP	3 days	Soup-Vegetable-Fruit DP<br>Eat out DP:<br>Traditional Chinese DP<br>Traditional Indian DP	SVF-DP inversely associated with anxiety;<br><br>TIC-DP: inversely associated with EPDS scores and ↓48% in probable depression
Avalos, 2020	**USA**	1160	**x**				**PHQ-9**	One time: 24W	Block 2005 modified (12)	One time: second trimester	Last 3 months	Poor diet quality defined as HEI-2010 scores in the lowest quartile Vs. Healthy Diet Quality	Women with prenatal depression Had:<br>↑ empty calories intake<br>↓ intake of greens and beans, total fruit, and whole fruit

Table S2. Consumption ranking, and percentile ranking, of the top 3 Fiber Food Groups in each Dietary Pattern & Simplified Mental Health outcomes.

Details of DP and High Fiber FGs					Consumption Ranking of Highest Fiber FGs in Each Dietary Pattern (%Rank)				Simplified Mental Health Outcomes For each DP		
**Study #**	**# of FGs/ Items**	**Top 3 Fiber FGs**	**DP**	**Name of DP**	**1st (Highest)**	**2nd Highest**	**3rd Highest**	**Average Ranking of Top3**	**Depression**	**Anxiety**	**Overall Mental Health**
Vilela, 2015& Vilela, 2014	19	Beans	DP1	Common Brazilian	2 (10.5)	8 (42.1)	5 (26.3)	5 (26.3)	0	(+)	(+)
		Fast Food & Snacks	DP2	Healthy	17 (89.5)	7 (36.8)	3 (15.8)	9 (47.4)	(+)	(+)	(+)
		Green Vegetables & Legumes	DP3	Processed	12(63.2)	3 (15.8)	15 (78.9)	10 (52.6)	0	0	0
Paskulin, 2017	62	Powdered Chocolate	DP4	Restricted	17 (27.4)	4 (6.5)	51 (82.3)	24 (38.7)		0	0
		Beans	DP5	Varied	44 (71.0)	5 (8.1)	41 (66.1)	30 (48.4)		Reference	0
		Lentils	DP6	Common Brazilian	56 (90.3)	4 (6.4)	45 (72.6)	35 (56.5)		(-)	(-)
Okubo, 2011	33	Seaweeds	DP7	Healthy	2(6.1)	3 (9.1)	5 (15.2)	3.3 (10.1)	0		0
		Mushrooms	DP8	Japanese	8(24.2)	14 (42.4)	7 (21.2)	9.7 (29.3)	(+)		(+)
		Beans	DP9	Western	19(57.6)	16 (48.5)	13 (39.4)	16 (48.5)	(+)		(+)
Miyake, 2018	33	Seaweeds	DP10	Healthy	5 (15.2)	3 (9.1)	4 (12.1)	4 (12.1)	(+)		(+)
		Mushrooms	DP11	Japanese	6(18.2)	16 (48.5)	3 (09.1)	8.3(25.3)	(+)		(+)
		Beans	DP12	Western	25 (75.8)	22 (66.7)	28 (84.8)	25 (75.8)	0		0
Miura, 2020	35	Natto	DP13	Unbalanced	25 (71.4)	34 (**97.1**)	17 (48.6)	25.3(72.4)	(-)		(-)
		Miso Soup	DP14	Japanese	24(68.6)	12 (34.3)	22 (62.9)	19.3(55.2)	(-)		(-)
		Chinese Noodles	DP15	Western	30(85.7)	16 (45.7)	23 (65.7)	23 (65.7)	0		0
	13	Beans	DP16	Unbalanced	9(69.2)	13 (100)	12 (92.3)	11.3(87.2)	(-)		(-)
		Vegetables	DP17	Japanese	6(46.2)	2 (15.4)	1 (7.7)	3 (23.1)	(-)		(-)
		Fruits	DP18	Western	7(53.8)	3 (23.1)	2 (15.4)	4 (30.8)	0		0
Teo, 2018	42	Seed Herbs	DP19	Traditional Chinese Confinement	Low intake	Low intake	Low intake	Low intake	0	0	0
		Legumes	DP20	Traditional Indian Confinement	4 (9.5)	7 (16.7)	1 (2.4)	4 (9.5)	(+)	0	(+)
		Ethnic Bread	DP21	Eat-Out	Low intake	Low intake	Low intake	Low intake	0	0	0
			DP22	Soup-Vegetable-Fruit	Low intake	Low intake	Low intake	Low intake	(+)	(+)	(+)
Cao, 2020	13		DP23	Beverage	10 (76.9)	11 (84.6)	5 (38.5)	8.7 (66.9)	0		0
			DP24	Vegetable	3 (23.1)	10 (76.9)	4 (30.8)	5.7 (43.6)	0		0
		Legumes	DP25	Cereals & Meats	13 (100)	10 (76.9)	6 (46.2)	9.7 (74.4)	0		0
		Nuts	DP26	Nuts & Fruits	4 (30.8)	1 (7.7)	2 (15.4)	2.3 (17.9)	(+)		(+)
		Fruits	DP27	Eggs	2 (15.4)	6 (46.2)	12 (92.3)	6.7 (51.5)	0		0
			DP28	Seafood	3 (23.1)	4 (30.8)	13 (100)	6.7 (51.5)	(+)		(+)
Huang, 2021	30		DP29	Varied	11 (36.7)	5 (16.7)	3 (10.0)	6.3 (21.1)	Reference		0
			DP30	Vegetable	12 (40)	9 (30.0)	4 (13.3)	8.3(27.7)	(+)		(+)
		Nuts	DP31	Meat	13 (43.3)	9 (30.0)	4 (13.3)	8.7 (28.9)	Symptoms =0; Scores=(+)		(+)
		Beans Products	DP32	Cereals	12 (40.0)	9 (30.0)	3 (10.0)	8 (26.7)	0		0
		Fruits	DP33	Milk	9 (30)	11 (36.7)	5 (16.7)	8.3 (27.7)	(+)		(+)
			DP34	Fruit	7 (23.3)	10 (33.3)	1 (3.3)	6 (20.0)	(+)		(+)
Baskin, 2017	33	Breakfast Cereal	DP35	Unhealthy	22 (66.7)	16 (48.5)	32 (96.7)	23.3 (70.7)	(-)		(-)
		Legumes<br>Nuts	DP36	Healthy	25 (75.8)	7 (21.2)	9 (27.2)	13.7 (41.4)	0		0
